# *MECP2* duplication syndrome in a Chinese family

**DOI:** 10.1186/s12881-015-0264-0

**Published:** 2015-12-16

**Authors:** Qingping Zhang, Ying Zhao, Yanling Yang, Xinhua Bao

**Affiliations:** Department of Pediatrics, Peking University First Hospital, No.1, Xi An Men Street, Xicheng District, Beijing, 100034 China

**Keywords:** *MECP2* duplication syndrome, Chinese family, MLPA, aCGH, Skewed X chromosome inactivation

## Abstract

**Background:**

Methyl-CpG-binding protein 2 (MeCP2) is a key transcriptional regulator of gene expression in the maintenance and development of the central nervous system. Loss- or gain-function of this gene may contribute to neurodevelopmental disorders. The aim of this study is to delineate the clinical characteristics of *MECP2* duplication syndrome and the hereditary mechanism in a Chinese family.

**Case presentation:**

We identified a Chinese family with three persons carry *MECP2* gene duplication: a boy, his mother and his grandmother. The duplication segment which was detected by multiplex ligation-dependent probe amplification (MLPA) included gene *MECP2*, interleukin-1 receptor-associated kinase 1 (*IRAK1*), filamin A (*FLNA*), and L1 cell adhesion molecule (*L1CAM*). Furthermore, array comparative genomic hybridization (aCGH) was performed on the mother, showed that *MECP2* containing duplication was 510 Kb (153,113,885-153,624,154), including 16 other genes except *MECP2*. The boy showed most symptoms of *MECP2* duplication syndrome. His mother and maternal grandmother were asymptomatic. Both female carriers had a skewed X chromosome inactivation (XCI), which were 80:20 and 74:26 respectively.

**Conclusion:**

To our knowledge, this is the second reported Chinese Han family with *MECP2*-containing duplications. And this patient had recurrent respiratory infections which was different from the first two Chinese-brother cases. *MECP2* is the core gene responsible for *MECP2* duplication syndrome. XCI may play an important role in modulating the clinical manifestation.

## Background

Methyl-CpG-binding protein 2 (*MECP2*) located at Xq28 acts as a transcriptional repressor or activator regulating the genes associated with nerve system development [1]. Loss-of-function mutations of *MECP2* gene are the cause of most cases of Rett syndrome (RTT, OMIM: 312750). RTT is a neurodevelopmental disorder which affects 1/10,000 girls. Recent studies found that duplications involving *MECP2* gene and increased *MECP2* protein dosage can cause a severe syndromic developmental delay (DD), mental retardation (MR), and recurrent infections. It was found in 1 % of patients with idiopathic X-linked MR (XLMR) and in 2 % of male patients with severe encephalopathy [2]. It was named *MECP2* duplication syndrome (*MECP2 DS*), a newly described genetic condition, and gain-of-function mutations of *MECP2*.

The cardinal features of the syndrome include early onset hypotonia, moderate to profound mental retardation, delayed psychomotor development, speech delay or absent to very limited speech, seizures, progressive spasticity in male patients. Dysmorphism is common and some features changed with age. Midface hypoplasia, depressed nasal bridge and a hypotonic face such as open mouth with tented upper lip, excessive drooling present in most patients at young age. When time goes by, some additional facial features may appear: a narrow nose, prominent chin, deep-set eyes, large ears, and widely spaced teeth [3]. Genital anomalies, including cryptorchidism and small penis, were reported in some cases. Patients with congenital central hypoventilation syndrome (CCHS), hyperkinesis were also reported [4, 5]. Half of these patients have a short lifespan less than 25-year-old [6]. The animal model with over expression both the mouse and human *MECP2* gene showed similar neurological symptoms including epilepsy, spasticity, hypoactivity and short lifespan. The severe phenotype is correlated with the higher Mecp2 protein level [7, 8].

Here we report a Chinese boy with *MECP2* duplication syndrome, who inherited the duplicated gene from his unaffected mother, while his mother inherited the duplicated gene from his unaffected grandmother. The genetic characters and the clinical features are presented.

## Case presentation

### Case report

The patient is a 2-year-4-month-old boy, who is the first child of his nonconsanguineous Chinese parents, a 26-year-old mother and a 27-year-old father, both of them are healthy. Following an uneventful pregnancy, he was born at 38 weeks of gestation with a birth weight of 3.15 kg. Feeding difficulties and poor suck had been noticed since birth. Hypotonia was apparent at age of 4 months. He showed severe developmental delay. He could not raise his head until 4 months old, sit unsupported till 11 months. At 2-year-4-month-old, he could not walk and speak any meaningful words. Eye contact and social smiling were enabling. He had recurrent respiratory tract infections, and hospitalized for severe pneumonia for several times. At 12 months old, his head circumference was 47 cm, weight was 11 kg. The boy had mild dysmorphism with flat nasal bridge, anteverted nares, small mouth, high-arched palate, low-set ears and cryptorchidism of the right side. Hand stereotypes including hand wringing and hand shaking presented at 8 months. He had no chronic constipation, no seizures. Magnetic resonance imaging (MRI) at age 1 year was unremarkable. Karyotype analysis was normal. Serum amino acid and urine organic acid screen were unremarkable.

**Fig. 1:**
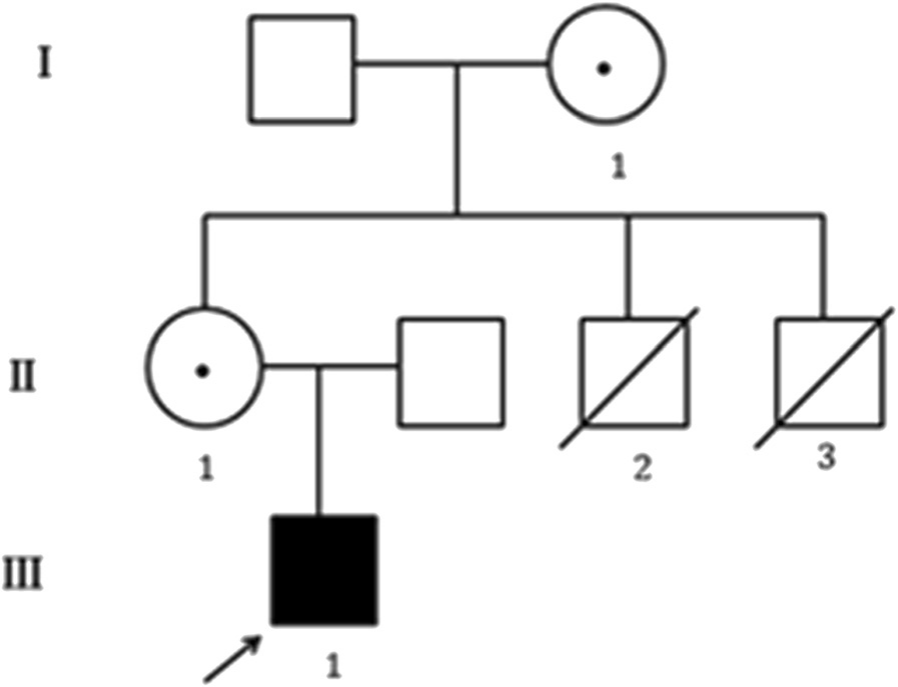
Pedigrees of the family. III-1 is the proband, I-1 and II-1 carry the *MECP2* duplication, II-2 and II-3 died at the age of 5-year-old and the age of 1y, respectively

Family history: The boy’s mother had two younger brothers. Both of them had severe developmental delay. The elder one could not walk or speak before he died at age of 5. The best motor function was sitting unaided. His brain computed tomography (CT) scan showed generalized cerebral atrophy. The younger one had a history of recurrent infections, died of a sever pneumonia at the age of 1y. He could not sit or walk alone during his life, speak no meaningful words yet. Both the mother and the grandmother were asymptomatic, they did not have any symptoms of depression, anxiety and compulsions. Figure 1 showed the pedigrees of the family, the proband was showed by an arrow.

### MLPA tests

Genomic DNAs from the peripheral blood leukocytes of the patient, his parents and the maternal grandmother were extracted using standard methods. MLPA (SALSA MLPA kit P015 *MECP2*, MRC-Holland, Amsterdam, Holland) was performed to detect large deletions or duplications of *MECP2* gene as previously described [9]. MLPA products were separated and analyzed using the ABI Prism 3100 Genetic Analyzer and Genescan software according to manufacturer’s recommendations.

### aCGH analysis

In order to determine the precise size of the duplication, aCGH was performed on the mother’ DNA, using Affymetrix GeneChip System 3000Dx v.2 (by Tianjin kingmed center for clinical laboratory, Shanghai, China).

### Fluorescence in situ hybridization (FISH)

FISH test was performed to interrogate the *MECP2* copy number and to identify the location of the duplicated *MECP2* gene in chromosome (done by Beijing Ahngook Pharm. Co., Ltd, Beijing, China).

### X chromosome inactivation patterns (XCI)

The patterns of XCI of female carriers were analyzed according to the procedure described by Allen et al. [10]. Inactivation was considered to be non- random if the ratio was > 70:30.

## Results

**Fig. 2:**
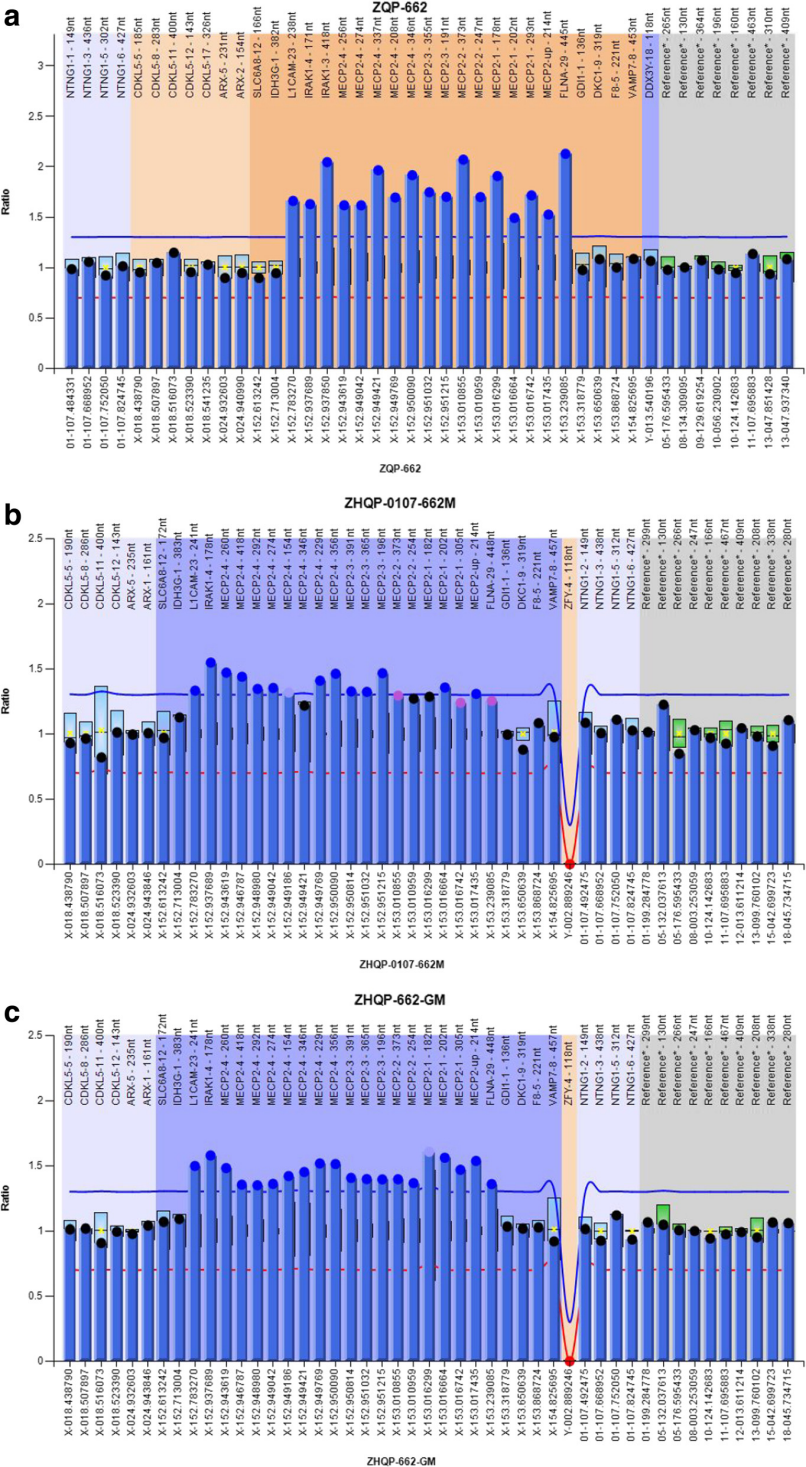
Genes duplication in the proband (**a**), his mother (**b**) and his grandmother (**c**), tested by MLPA

DNA fragment duplication was found in the patient, his mother and maternal grandmother by MLPA test. The fragment range from X-152,783,270 to X-153,239,085, including L1 cell adhesion molecule gene (*L1CAM*; OMIM #308840), interleukin-1 receptor-associated kinase 1 (*IRAK1*; OMIM #300283), *MECP2*, filamin A (*FLNA*; OMIM #300017) and other genes, that probes were not designed in the kit P015 (Fig. 2).

aCGH was performed on the proband’s mother to test the precise size of the duplicated fragment. The result showed that the duplication fragment was 510, 270 bps (153,113,885–153,624,154) containing 17 genes (Table 1), including two subunits of *Micro-RNA (MIR3202)*, starting from *L1CAM* and ending with emerin *(EMD*; OMIM #300384*)*. There was no gene coming across the breakpoints.

**Table 1:** Duplicated genes and their positions

Gene	Position
L1 cell adhesion molecule *(L1CAM)*	X:153,126,969–153,141,500
Arginine vasopressin receptor 2 *(AVPR2)*	X:153,170,428–153,172,620
Rho GTPase activating protein 4 *(ARHGAP4)*	X:153,172,830–153,191,714
N (alpha)-acetyltransferase 10 *(NAA10)*	X:153,195, 280–153,200,607
Renin binding protein *(RENBP)*	X:153,200,722–153,210,232
Host cell factor C1 *(HCFC1)*	X:153,213,008–153,236,819
Transmembrane protein 187 *(TMEM187)*	X:153,237,991–153,248,646
MicroRNA 3202–1 *(MIR3202-1* ^*a*^ *)*	X:153,246,548–153,246,628
MicroRNA 3202–2 *(MIR3202-2* ^*a*^ *)*	X:153,246,549–153,246,627
Interleukin-1 receptor-associated kinase 1 *(IRAK1)*	X:153,275,957–153,285,342
MicroRNA 718 *(MIR718)*	X:153,285,371-153,285,440
Methyl CpG binding protein 2 *(MECP2)*	X:153,287,264–153,363,188
Opsin 1 (cone pigments), long-wave-sensitive *(OPN1LW)*	X:153,409,725–153,424,507
Opsin 1 (cone pigments), medium-wave-sensitive *(OPN1MW)*	X:153,485,203–153,499,470
Testis expressed 28 *(TEX28)*	X:153,498,929–153,523,563
Transketolase-like 1 *(TKTL1)*	X: 153,524,027–153,558,713
Filamin A, alpha *(FLNA)*	X:153,576,900–153,603,006
Emerin *(EMD)*	X:153,607,597–153,609,883

^a^Two subunits of micro-RNA3202

**Fig. 3:**
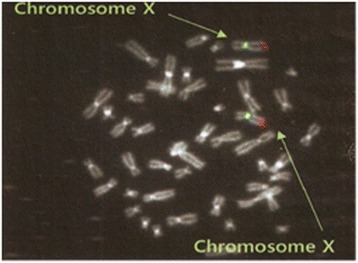
Two-color fluorescence in situ hybridization analysis of the mother with *MECP2* gene duplication. The duplicated *MECP2* gene was located at X chromosome, and only one fluorescence signal can be seen on each X chromosome. Green signal is a control single-copy probe, hybridized to alpha satellite DNA at the chromosome X centromere. Red signal on distal Xq is a probe for the *MECP2* gene, which is present on each X chromosome

FISH test was performed on the mother. The red fluorescence signals representing *MECP2* gene were seen on each X chromosome, and only one fluorescence signal could be seen on each X chromosome (Fig. 3). That means the duplicated *MECP2* gene was located at X chromosome next to the original *MECP2* gene.

The XCI pattern analysis showed the XCI ratio of the patient’s mother and maternal grandmother was 80:20 and 74:26, respectively. The X chromosome carrying the duplication was 80 % inactive in the mother and 74 % inactive in the grandmother. The proband inherited the X chromosome carrying the duplication variant from his mother.

## Discussion

With the development of new techniques, some genomic disorders such as Williams-Beuren syndrome (WBS, MIM 194050), Potocki-Lupski syndrome (PTLS, MIM 610883), Miller-Dieker syndrome (MDS, MIM 247200), were found to be caused by the alteration of gene dosage, or copy number variation (CNV) [11]. *MECP2* gene acting as a transcriptional regulator of gene expression should express very accurate level in cells. Loss-of-function of *MECP2* leads to Rett syndrome while gain-of-function causes *MECP2* duplication syndrome. In additional, patient with Xq28 rearrangement containing *MECP2* triplication was considered to be more severe than *MECP2* duplication [12, 13]. Animal models also demonstrated this phenomenon.

*MECP2* duplication is most common among the Xq28 rearrangement. The reported duplications differed in size and location (except the carrier mom and inherited offspring), were defined as non-recurrent events. The flanking region around *MECP2* gene is abundant of low-copy repeat (LCR) sequences and high GC content, which generated the genomic instability such as double-strand breaks (DSBs) and a collapsed fork. The molecular mechanisms of *MECP2* copy number variation were proposed by mainly two ways: one is Fork Stalling and Template Switching (FoSTeS), another is microhomology mediated break-induced replication (MMBIR) [14–17].

In the previous reports, the sizes of the duplications encompassing *MECP2* locus vary between 79.7 kbs and 15.5 Mbs [18, 19]. The smallest duplication only contains *MECP2* and *IRAK1* genes. The duplication size in our patient was about 510 kbs, including 17 genes, starting from *L1CAM* and ending with *EMD. L1CAM, IRAK1, MECP2* and *FLNA* were embraced in the duplication. These four genes play important roles in postnatal nervous system development. *L1CAM* and *MECP2* were associated with X-linked mental retardation (XLMD). *IRAK1*, encoding an interleukin receptor–associated kinase was involved in the toll–like receptor transduction pathway, was a critical signaling mediator of innate immunity. *Gottipati* et al. found that over expression of this gene might lead to the susceptibility to infections [20]. However, *Yang* and colleagues identified immune defects among the patients with *MECP2* DS had no association with *IRAK1* duplication by transgenic mice that overexpressed *MECP2* without *IRAK1* duplication [21]. This conclusion was supported by *Sawalha* [22]. Chronic intestinal pseudo–obstruction (CIPO), thrombcytopaenia, patent ductus arteriosus (PDA), malrotation were found in two families with the duplications involved only the *FLNA* gene [23]. One case suffered CCHS also had chronic constipation without duplication of *FLNA* [24]. Some other genes in the duplicated area of our patient may be associated with mental retardation and development delay. *Huang* et al. reported 2 male-dizygotic-twins with a 17.9-kbs deletion of Xq28 including Arginine vasopressin receptor-2 gene (*AVPR2*; OMIM 300538) and extending into intron 7 of the RHO GTPase-activating protein 4 gene (*ARHGAP4*; OMIM 300023). They were diagnosed with nephrogenic diabetes insipidus (NDI) as well as psychomotor developmental delay [25]. Host cell factor C1 gene (*HCFC1;* OMIM 300019) was reported to be associated with X-linked mental retardation (XLMR) [26]. The clinical manifestation of our patient was characterized by developmental delay, early hypotonia, recurrent infection, and mild dysmorphism, without CIPO, PDA and NDI. Combined our study and previous reports, most of the symptoms of *MECP2 DS* should be attributed to gain of MeCP2 function.

Family study showed the mother and maternal grandmother also carried the *MECP2* duplication. The patient inherited the mutation from them. FISH test on the mother showed the duplicated *MECP2* gene was located at X chromosome. However, the mother and the maternal grandmother were asymptomatic. Further XCI study showed the XCI pattern was non-random in both of them. The ratio was 80:20 and 74:26 respectively. The X chromosome carrying the duplication was prioritized inactive. This molecular phenomenon may explain their asymptomatic phenotype. In the previous reports, almost all the asymptomatic carriers were with a skewed XCI except one with XCI ratio 43:57 [27]. However, the XCI ratio in peripheral blood DNA may not represent the ratio in Brain. It could be inconsistent in different tissues. A mother of a *MECP2* DS patient had mild learning difficulties with a complete skewed XCI in the blood DNA while the ratio was 74:26 in her hair roots. The authors speculated that the inactivation of X chromosome with *MECP2* duplication in her brain was not completely leading to her mild clinical features [28]. This is the only literature about XCI test performed on the other tissue rather than blood in patients with *MECP2* DS. In this family, two maternal uncles of the boy had severe developmental delay, severe mental retardation and recurrent infections. Both of them died at young age. They were highly suspected to be patients with *MECP2* DS.

## Conclusions

In this study, we demonstrate one more Chinese family with *MECP2* duplication syndrome. The main clinical features include early onset hypotonia, mental retardation, recurrent respiratory tract infections, limited speech and dysmorphism. Female carrying the *MECP2* duplication could be asymptomatic due to the skewed XCI. In order to make an accurate diagnosis and provide a right genetic counseling, genomic structure rearrangement, especially *MECP2* duplication should be tested in the patients with the above symptoms as well as other family members.

## Consent

Gene mutational analysis on the family members and the study of the XCI patterns in female carriers began after signing an informed consent by the parents. Ethical approval was obtained from the hospital research ethic board. Written informed consent was obtained from the legal guardian of the patient for publication of this Case report and any accompanying images. A copy of the written consent is available for review by the Editor of this journal.
